# Annual Feedback Is an Effective Tool for a Sustained Increase in Calcium Intake among Older Women

**DOI:** 10.3390/nu20901018

**Published:** 2010-09-17

**Authors:** Kerrie M. Sanders, Amanda L. Stuart, Mark A. Kotowicz, Geoffrey C. Nicholson

**Affiliations:** Department of Clinical and Biomedical Sciences, Barwon Health, The University of Melbourne, Victoria 3220, Australia; Email: amandh@barwonhealth.org.au (A.L.S.); markk@barwonhealth.org.au (M.A.K.); geoffn@barwonhealth.org.au (G.C.N.)

**Keywords:** calcium intake, annual feedback, older women, fractures

## Abstract

We aimed to optimize calcium intake among the 2,000+ older women taking part in the Vital D study. Calcium supplementation was not included in the study protocol. Our hypothesis was that annual feedback of calcium intake and informing women of strategies to improve calcium intake can lead to a sustained increase in the proportion of women who consume adequate levels of the mineral. Calcium intake was assessed on an annual basis using a validated short food frequency questionnaire (FFQ). Supplemental calcium intake was added to the dietary estimate. Participants and their nominated doctor were sent a letter that the participant’s estimated daily calcium intake was adequate or inadequate based on a cutoff threshold of 800 mg/day. General brief statements outlining the importance of an adequate calcium intake and bone health were included in all letters. At baseline, the median daily consumption of calcium was 980 mg/day and 67 percent of 1,951 participants had calcium intake of at least 800 mg per day. Of the 644 older women advised of an inadequate calcium intake at baseline (<800 mg/day), 386 (60%) had increased their intake by at least 100 mg/day when re-assessed twelve months later. This desirable change was sustained at 24 months after baseline with almost half of these women (303/644) consuming over 800 mg calcium per day. This study devised an efficient method to provide feedback on calcium intake to over 2,000 older women. The improvements were modest but significant and most apparent in those with a low intake at baseline. The decreased proportion of these women with an inadequate intake of calcium 12- and 24-months later, suggests this might be a practical, low cost strategy to maintain an adequate calcium intake among older women.

## 1. Introduction

The lifetime risk of sustaining a fragility fracture is attenuated by an adequate calcium intake throughout life with a particular focus during old age when the risk of fracture is highest. The estimated average requirement (EAR) for calcium is highest for this older sector of our population corresponding to a time when total energy needs are reduced and appetite may be compromised by concurrent medications, co-morbidities and poor mobility. The average Australian woman aged at least 70 does not meet the estimated requirement for calcium intake [1] and long-term adherence to calcium supplements is typically poor [2,3,4,5,6,7]. Thus, ensuring adequate intake in older women is a challenge.

We aimed to optimize calcium intake among the 2,000+ older women taking part in the Vital D study. This randomized controlled trial investigated whether a single annual dose of cholecalciferol reduced falls and fractures in older women. Calcium supplementation was not included in the study protocol. Our hypothesis was that annual feedback of calcium intake and informing women of strategies to improve calcium intake can lead to a sustained increase in the proportion of women who consume adequate levels of the mineral.

## 2. Methods

This analysis is part of a randomized, double-blind placebo-controlled study investigating whether a single annual dose of cholecalciferol (500,000 IU vitamin D_3_) administered orally to older women in autumn or winter would reduce the risk of falls and fracture [8]. The study was a pragmatic design so that findings of benefit could be easily translated to clinical practice. Older women could theoretically receive their annual oral dose of vitamin D each autumn while visiting their general practitioner for the influenza vaccination. Since calcium supplements need to be taken on a daily basis, this was not included as part of the study protocol. However participants’ calcium intake was calculated at baseline and re-assessed on an annual basis for four to six years. Participants were sent a letter that their estimated daily calcium intake was adequate or inadequate based on a cutoff threshold of 800 mg/day [9]. General brief statements outlining the importance of an adequate calcium intake and bone health were included in all letters. The correspondence included advice that their nominated doctor had been advised of their calcium intake. A pamphlet from the dairy board was also included as well as a statement regarding supplements if the participant considered that they could not adequately increase their calcium intake through diet. Awareness of the importance of an adequate calcium intake was highlighted in the study newsletter sent out to all participants twice a year.

Calcium intake was assessed annually using a validated short food frequency questionnaire (FFQ) [10]. Respondents were asked to select the type of milk they habitually used and the number of tea and coffee drinks they consumed per day. The twelve items listed with standard serve quantities given on the table were: Milk on cereal; milk as a drink; milk in tea and coffee; milk in home cooked foods such as custard; hard cheeses; soft cheeses; yoghurt or other dairy or soy dessert; ice cream; three types of canned fish and spinach or silverbeet. Respondents needed to complete the number of serves per week for each of the 12 food items. It was assumed these items collectively contribute 60 percent of the daily dietary calcium intake. The questionnaire was modified to include more recently developed food products such as specialized milks that provide a significant source of calcium. Calcium content was taken from the product information labeling and Australian food composition data [11]. A spreadsheet with imbedded formulae was developed so that participants’ responses to the closed questions on the calcium FFQ were electronically entered and the calcium daily intake automatically generated. Medications and use of dietary supplements were concurrently recorded at each annual assessment and included in the estimate of calcium intake. Each year participants were also asked if they considered they had changed their calcium intake since the previous assessment one year ago. Our reporting of changes in calcium intake refers to the re-assessment 12 months after receiving feedback and literature on calcium intake.

### Statistics

Calcium intakes were estimated in milligrams per day and then categorized into one of the following four groups based on clinical significance of calcium intake in relation to bone health: (1) less than 800 mg/day; (2) 800 to 1,100 mg/day; (3) 1,100 to 1,300 mg/day and (4) greater than 1,300 mg/day. Calcium intake was considered to have changed if the estimated daily intake differed from a prior estimate by at least 100 mg/day. Estimated intakes per day have been rounded to the nearest 10 mg reflecting the margin of error in individuals’ self-report of food frequency intakes. Results were analyzed using cross tabulation and Chi square testing using Minitab™ version 15. 

The study was approved by the Barwon Health Human Research Ethics Committee (BH HREC 02/60) and The University of Melbourne Human Research Ethics Committee (HREC No. 0200700). All participants provided written informed consent to participate. The study is registered with the Australian Clinical Trial Registry (ACTR No. 12605000658617) and the International Standard Randomized Controlled Trial Number Register (ISRCTN No. 83409867).

## 3. Results

Over two years of recruitment from 2003 to 2005, 2,317 community dwelling women aged at least 70 years consented to participate in this randomized controlled trial [12]. A total of 226 participants withdrew from the study before the pre-determined study end in 2008. The main reasons for withdrawal included death (n = 87), illness (n = 52), disinterest (n = 31), age or dementia (n = 21), personal reasons (n = 14) or other (n = 21). For the purpose of this analysis allocation to treatment group is ignored since both participants and researchers were blinded to treatment status (vitamin D_3_ or placebo) throughout the entire period of calcium estimation and there was no difference in category of baseline calcium intake between treatment groups (p = 0.42). The analysis of change in calcium intake over three consecutive years is restricted to 1,951 participants for whom we have complete data. Participants were aged between 70 and 91 with a median age of 76 years.

At baseline, the median consumption of calcium was 980 mg/day (Table 1). The proportion of the total cohort with an inadequate intake (less than 800 mg/day) decreased from 33 percent at baseline to approximately 28 percent during Years two and three. Thus approximately 47% of those having an inadequate intake at baseline were consuming an adequate calcium (greater than 800 mg) intake by Year 3. Most of this positive change occurred in the first 12-month period followed by a maintenance of this increase during the 12 to 24-month period (Table 2: median increase 170 mg/day and 10 mg/day, respectively). Correspondingly those having 1,300 mg/day or more increased from 26 percent to 32 percent. The proportion of women taking calcium supplements increased from 18 percent at baseline to 22 percent at Year two and 25 percent at Year three (Table 2). This represents the total proportion of participants taking calcium supplements at these time points despite, 17 percent of the women taking calcium supplements at baseline having ceased the supplementation twenty-four months later (n = 60/348). Forty-one percent (n = 804/1951) of participants had a calcium intake between 800 and 1300 mg/day and this proportion of women remained stable over the three annual assessments.

**Table 1:** Estimated calcium intake (mg/day) and proportion of women in each category of intake.

	Median	IQR		<800	800–1099	1100–1299	1300+
				Percent	Percent	Percent	Percent
Year 1	980	690	1,310	33 (644)	27 (530)	14 (274)	26 (503)
Year 2	1,070	760	1,410	28 (548)	25 (490)	15 (294)	32 (619)
Year 3	1,080	780	1,410	27 (524)	24 (472)	17 (340)	32 (615)

Percent refers to percentage of 1,951 participants in each category of daily calcium intake; parentheses refer to the actual number of participants; IQR = Inter Quartile Range.

**Table 2:** Change in dietary calcium intake in those above and below 800mg intake/day at baseline.

	Baseline calcium intake <800 mg/day, n = 644	Baseline calcium intake ≥800 mg/day, n = 1,307	Total Group n = 1,951
**Daily calcium intake <800 mg/day at:**			
Baseline			**33% (644)**
Year 2	57% (367)	14% (181)	**28% (548)**
Year 3	53% (340)	14% (184)	**27% (524)**
**Calcium supplements users:**			
Baseline	3% (18)	25% (330)	**18% (348)**
Year 2	15% (94)	26% (339)	**22% (433)**
Year 3	17% (110)	29% (373)	**25% (483)**
**Change in daily calcium intake over 12-month period between:**			
Baseline and Year 2	170 mg [0, 450]	−10 mg [−240, 240]	**60 mg [−160, 310]**
Year 2 and Year 3	10 mg [−190, 220]	−20 mg [−250, 220]	**0 mg [−230, 220]**
**Increase in daily calcium intake by diet:**			
Year 2	320 mg [190, 500]	300 mg [175, 445]	**300 mg [270, 840]**
Year 3	270 mg [180, 420]	290 mg [179, 434]	**280 mg [180, 430]**
**Increase in daily calcium intake by supplement:**			
Year 2	750 mg [490, 1130]	400 mg [230, 700]	**510 mg [270, 840]**
Year 3	470 mg [270, 740]	390 mg [230, 720]	**420 mg [240, 720]**
**Participants who claimed to increase daily calcium intake:**			
Year 2	24% (157)	11% (148)	**16% (305)**
Year 3	14% (92)	11% (149)	**12% (241)**
**Participants who claimed to increase and did increase their daily calcium intake:**			
Year 2	87% (137)	55% (82)	**72% (219)**
Year 3	58% (53)	46% (69)	**51% (122)**

Of the women who increased their calcium intake, the median increase through diet alone was 300 mg/day (IQR: 180, 470 mg/day; n = 607) and through supplements was 510 mg/day (IQR: 270, 840 mg/day; n = 271) at 12 months. For the following 12-month period (12 to 24 months) the median increase through diet was 280 mg/day (IQR: 180, 430 mg/day; n = 502) and 420 mg/day (IQR: 240, 720 mg/day; n = 220) through supplements.

At baseline only 3 percent of those with an inadequate intake were taking calcium supplements (Table 2) and most were not taking the tablets on a daily basis (n = 11/18). Fifteen percent (n = 94/644) were taking calcium supplements by Year two and 17 percent (n = 110/644) during Year three. Of those categorized at baseline as having inadequate intake (n = 644), 60 per cent had increased their intake by at least 100 mg/day when re-assessed twelve months later and almost half (47%) had increased their intake to be at least 800 mg/day twenty-four months later. The largest incremental increase (750 mg/day) occurred in those who commenced supplements after being notified of an inadequate calcium intake at baseline.

When participants were asked at 12-months if they had changed their calcium intake since baseline, 24% of those originally classified as ‘inadequate’ responded that they had improved their calcium intake compared to 11% of those with an adequate baseline intake (p < 0.001, Table 2). Of these participants 87% were successful and had increased their calcium intake by at least 100 mg/day. 

## 4. Discussion

The results suggest annual feedback advice relating to adequacy of total calcium intake provided to both the individual and their doctor is an effective strategy to increase calcium in those with an inadequate intake. Of the 644 older women who were advised of an inadequate calcium intake at baseline (<800 mg/day), 60 per cent had increased their intake by at least 100 mg/day when re-assessed twelve months later. This desirable change was sustained at 24 months after baseline with almost half of these women now having an intake over 800 mg/day. 

In Australia the estimated average requirement (EAR) for calcium for women over 50 years is 1,100 mg/day with the recommended amount being 1,300 mg/day. To achieve this intake it is recommended to have at least 3 serves of dairy per day with at least one of those being calcium fortified [13]. High calcium foods are often also a rich source of high quality protein as well as other vitamins and minerals which help maintain general health and improve immunity in the elderly [14]. Despite these potential benefits many older women are unable to maintain a daily intake of 1,300 mg. Our minimum adequate threshold for calcium intake is derived from results of an RCT involving 1,363 older Australian women reporting a threshold for bone health of 792 mg/day [9]. This threshold is supported by findings that calcium intake above 800 mg/day may be unnecessary as long as serum 25-hydroxyvitamin D exceeds 45 nmol/L [15]. Although not a consistent finding, the skeletal benefit of increasing calcium intakes is generally regarded as greatest in those with lower baseline calcium intakes [3,13]. On this basis those most likely to benefit, *i.e.*, women with low intake, are the group that increased their calcium intake following the annual feedback of an inadequate calcium intake. By the second and third annual assessments there were fewer women in the lowest category of intake (inadequate intake <800 mg/day) and higher proportion of women were regularly having at least 1,300 mg/day compared to baseline.

The proportion of women who had ceased taking their calcium supplement 24 months later (17%) is substantially lower than the 30 to 45 percent commonly reported in randomized controlled trials [2,3,5,6,7]. The women taking supplements in our study were doing so as their own choice and not as part of a study protocol. Nevertheless the higher rate of adherence is noteworthy. Increased habitual calcium intake has been shown to lower bone turnover markers [16]. Although the beneficial effect of calcium supplementation is most marked in the first year of treatment, a slower, more cumulative effect is thought to occur in cortical bone [17,18].

In an 8 week study of 80 females aged 19 to 30 years, an educational intervention to increase dietary intake of calcium and vitamin D did not result in a significant change in calcium intake despite an inadequate intake at baseline [19]. Our results may differ due to the different age of participants between the two studies. The risk of fragility fracture obviously represents more real threat among the Vital D participants who were all aged at least 70 years and may have already experienced a fracture in adulthood. Additionally our education was focused on increasing calcium intake rather than awareness of osteoporosis and participants were notified that their doctors had also been advised of their calcium intake.

The strength of the study is the length of follow-up and the large number of participants. The estimation of calcium using the shortened food frequency questionnaire was easy for the older women to complete. Contact with participants was done entirely by post and telephone for those unable to access a post box. Although participants were asked if they had changed their calcium intake since the last assessment, reasons for change were not documented. A limitation of the study was that there was no comparative group that did not receive the annual feedback on calcium intake. Another limitation relates to the estimate of nutrient intakes. Food frequency questionnaires are designed to assess intake of specific nutrients in large number of individuals. Although the calcium FFQ used in this study has been previously shown to have a good correlation between weighed food record (r = 0.79) [10], participants given an indication of adequacy rather than the actual estimate of daily calcium which may otherwise have given a false impression of precision. 

## 5. Conclusions

Previous studies have stated that the moderate to low compliance of daily calcium supplements is likely to limit the effectiveness of calcium supplementation as a public health intervention to combat fragility fractures in the elderly [3,20]. This study devised an efficient method to provide feedback on calcium intake to over 2,000 older women. It does not relate only to supplementation but total calcium intake. The improvements were modest but significant and most apparent in those with a low intake at baseline. The decreased proportion of these women with an inadequate intake of calcium 12- and 24-months later, suggests this might be a practical, low cost strategy to maintain an adequate calcium intake among older women.
